# Psychiatric Co-occurring Symptoms and Disorders in Young, Middle-Aged, and Older Adults with Autism Spectrum Disorder

**DOI:** 10.1007/s10803-016-2722-8

**Published:** 2016-02-09

**Authors:** Anne G. Lever, Hilde M. Geurts

**Affiliations:** Dutch Autism and ADHD Research Center, Department of Psychology, University of Amsterdam, Nieuwe Achtergracht 129B, 1018 WT Amsterdam, The Netherlands; Dr. Leo Kannerhuis, Research Development and Innovation, Houtsniplaan 1a, 6865 XZ Doorwerth, The Netherlands

**Keywords:** Autism spectrum disorder, Psychiatric comorbidity, Aging, Adults, Depression, Anxiety

## Abstract

Although psychiatric problems are less prevalent in old age within the general population, it is largely unknown whether this extends to individuals with autism spectrum disorders (ASD). We examined psychiatric symptoms and disorders in young, middle-aged, and older adults with and without ASD (N_max_ = 344, age 19–79 years, IQ > 80). Albeit comparable to other psychiatric patients, levels of symptoms and psychological distress were high over the adult lifespan; 79 % met criteria for a psychiatric disorder at least once in their lives. Depression and anxiety were most common. However, older adults less often met criteria for any psychiatric diagnosis and, specifically, social phobia than younger adults. Hence, despite marked psychological distress, psychiatric problems are also less prevalent in older aged individuals with ASD.

**Electronic supplementary material:**

The online version of this article (doi:10.1007/s10803-016-2722-8) contains supplementary material, which is available to authorized users.

## Introduction

Psychopathology is a frequently occurring phenomenon. In the general population, approximately 40 % meets criteria for a psychiatric disorder at least once in their lives (Bijl et al. 1998; Kessler et al. 2005). This rate is much higher in individuals with an autism spectrum disorder (ASD), a heterogeneous neurodevelopmental disorder characterized by atypicalities in social communication and interaction and repetitive stereotyped behavior (American Psychiatric Association 2013). In this population, at least 69 % is thought to suffer from co-occurring psychiatric disorders and symptoms (Buck et al. 2014), even though rates are lower in individuals with ASD and intellectual disability (ID) (Howlin and Moss 2012; Matson and Cervantes 2014). The presence of co-occurring disorders is associated with lower quality of life, greater demands for professional help, poorer prognosis, greater interference with everyday life, and worse outcome (Lainhart 1999; Matson and Cervantes 2014; Seltzer et al. 2004; Vannucchi et al. 2014; Wood and Gadow 2010). Furthermore, specifically the co-occurring symptoms and disorders often constitute a target for treatment, leading to an amelioration of problems. For example, various psychotropic medications are frequently prescribed to individuals with ASD to treat associated symptoms (Aman et al. 2005; Buck et al. 2014; Esbensen et al. 2009; Logan et al. 2015; Seltzer et al. 2004). As ASD is considered a lifelong disorder (Piven et al. 1996; Seltzer et al. 2004) and symptoms of psychopathology are likely to wax and wane across the adult lifespan, knowledge regarding associated psychopathology in older adulthood is needed (Matson and Cervantes 2014; Perkins and Berkman 2012) to be able to provide adequate support for these older individuals. This will be the focus of the current study.

In the general population, age is a relevant factor for psychopathology. The prevalence of psychiatric disorders and their nature is different in older adulthood than in middle or young adulthood (Bijl et al. 1998; Kessler et al. 2005). While the general prevalence of psychiatric disorders is lower, the prevalence of, for example, alcohol or substance related disorders decreases sharply with increasing age, whereas depression and anxiety are still highly prevalent (Beekman et al. 1998; Wolitzky-Taylor et al. 2010).

While traditionally many ASD studies mainly focused on co-occurring symptoms and disorders in childhood (de Bruin et al. 2007; Leyfer et al. 2006; Lundström et al. 2015; Mattila et al. 2010; Mukaddes et al. 2010; Simonoff et al. 2008; Sinzig et al. 2009; van Steensel et al. 2013; Verheij et al. 2015), recently a steadily increasing number of studies have taken into account co-occurring symptoms and disorders in adulthood (Buck et al. 2014; Cervantes and Matson 2015; Croen et al. 2015; Ghaziuddin and Zafar 2008; Hofvander et al. 2009; Joshi et al. 2013; Lugnegård et al. 2011; Maddox and White 2015; Roy et al. 2015). These findings seem to suggest that also in the ASD population age is an important factor. In childhood, attention deficit hyperactivity disorder (ADHD), behavioral disorder, and anxiety disorders are the most prevalent comorbid disorders (de Bruin et al. 2007; Leyfer et al. 2006; Simonoff et al. 2008; Sinzig et al. 2009), whereas in adulthood, next to ADHD and anxiety disorders, mood disorders are common (Croen et al. 2015; Ghaziuddin et al. 2002; Ghaziuddin and Zafar 2008; Hofvander et al. 2009; Joshi et al. 2013; Roy et al. 2015; Sterling et al. 2008). In various adult studies, older adults with ASD have been included but only a few directly compared older adults with younger individuals (Roy et al. 2015; Totsika et al. 2010). In an intellectually challenged (ID) sample, psychiatric disorders were less frequent in older adults with ASD and ID compared to younger adults with ASD and ID (Totsika et al. 2010). In contrast, in older adults with ASD without ID, co-occurring psychiatric disorders were more common than in younger adults (Roy et al. 2015). Unfortunately, the “older group” in this latest study was relatively young (age range 40–62 years), the sample was small, and a statistical comparison was lacking. A few studies focused on specific psychiatric disorders such as anxiety (Davis et al. 2011) and depression (Ghaziuddin et al. 2002). Whereas anxiety seemed to reduce from childhood to young adulthood (Davis et al. 2011), the risk for depression seemed to increase with increasing age (Ghaziuddin et al. 2002). A small study in older adults (53–83 years) with ASD reported high levels of psychological and somatic complaints and of psychological distress (van Heijst and Geurts 2014). However, it has not been tested whether these participants encountered a sufficient number of psychiatric symptoms to meet diagnostic criteria, although also associated symptoms in itself may cause clinically relevant distress and impairment that may interfere with quality of life and daily functioning. Thus, the nature and prevalence of comorbid psychopathological symptoms and disorders in older adults with ASD is largely unknown. In the current study, we will, therefore, determine both the occurrence of non-ASD symptomatology and co-occurring psychiatric disorders across the adult lifespan in ASD by comparing young, middle-aged, and older adults clinically diagnosed with ASD without ID. We hypothesize psychiatric co-occurring symptoms and disorders to be substantially higher in individuals with ASD than in controls over the whole adult lifespan, but comparable to a normative group of policlinic psychiatric patients (Joshi et al. 2013). Given the mixed findings so far (Davis et al. 2011; Roy et al. 2015; Totsika et al. 2010), we will examine whether there will be differences in this co-occurrence of other psychiatric symptoms and disorders between the three age groups.

In addition to age, several other factors might affect the prevalence of comorbid psychiatric disorders in individuals with ASD, including ASD severity, gender, social economic status (i.e., education and work), living situation, and both intellectual and more general cognitive functioning. We will explore their role with respect to the co-occurring psychopathology in adults with ASD. For example, in the general population vulnerability factors for developing anxiety or depression are, among others, cognitive decline, being female, having a lower social economic status, or not having a partner (Beekman et al. 1998). In the ASD literature the focus has been mainly on ASD severity, gender, and intellectual functioning but whether these factors are indeed risk factors for comorbid psychopathology in ASD is a topic of debate as results are rather inconsistent (Cederlund et al. 2010; García-Villamisar and Rojahn 2015; Gotham et al. 2015; Holtmann et al. 2007; Jang and Matson 2015; Lai et al. 2011; Lugnegård et al. 2011; Moss et al. 2015; Simonoff et al. 2008, 2013; Sterling et al. 2008; Tureck et al. 2014; van Steensel et al. 2012; Verheij et al. 2015). To restrict the number of analyses we will solely explore whether all these aforementioned factors are indeed risk factors correlated with the most commonly co-occurring disorders in adults with ASD, which we expect to be mood and anxiety disorders (see for a similar approach in children Simonoff et al. 2008, 2013).

## Methods

### Participants

Two-hundred-forty-seven adults with ASD between 19 and 79 years were recruited through several mental health institutions across the Netherlands and by means of advertisements on client organization websites. Individuals with ASD traits, but without a prior clinical diagnosis of ASD based on DSM-IV criteria (autism, Asperger’s syndrome, and Pervasive Developmental Disorder Not Otherwise Specified) (American Psychiatric Association 2000), which was generally diagnosed by a multidisciplinary team involving a psychologist and/or psychiatrist,^1^ were not eligible to participate in the study.

Two-hundred-eight adults without ASD [comparison (COM) group] were recruited by means of advertisements on the university website and on social media, and within the researchers’ social environment. Individuals with a considerable amount of autistic traits, as measured with the Autism-spectrum Quotient (AQ > 32) (Baron-Cohen et al. 2001), or with close family members having ASD or schizophrenia, were excluded.

For both groups, additional requirement upon participation was an absent history of neurological disorders (e.g., epilepsy, stroke, cerebral contusion) or schizophrenia. Four-hundred-five individuals met these prerequisites (216 ASD, 189 COM). The study consisted of two parts. Part I included the administration of a questionnaire on psychological symptoms and distress and medication usage, which was completed by 344 individuals (172 ASD, 172 COM) who constituted the final sample of Part I. Part II included the administration of a neuropsychiatric interview to examine psychiatric disorders and an analysis of potential risk factors, and was part of a larger study assessing age-related differences in cognition (Lever & Geurts 2015a). Eligible ASD individuals were selected based on age to ascertain that participants were evenly distributed across ages. IQ was estimated with two subtests of the Dutch Wechsler Adult Intelligence Scale third edition (WAIS-III) (Uterwijk 2000; Wechsler 1997) and the diagnoses of the ASD participants were verified by administering the Autism Diagnostic Observation Schedule module 4 (ADOS) (de Bildt and de Jonge 2008; Lord et al. 2000). Four individuals (2 ASD, 2 COM) had an estimated IQ below 80 and were excluded from the sample of Part II. Of the remaining 138 ASD participants, 37 scored below the ADOS cut-off for ASD (<7), 49 below the autism threshold (<10), and 52 above the autism threshold (≥10). As all these individuals had a clinical diagnosis within the autism spectrum, diagnosed independently from the present study by mental health professionals, and the concordance between clinical diagnosis and ADOS module 4 classification is not unequivocal in intellectually able individuals (Bastiaansen et al. 2011a; Pugliese et al. 2015; Ring et al. 2015), we included all these ASD participants in the current study. Furthermore, 80 % scored above the threshold of 26 on the AQ (Woodbury-Smith et al. 2005). All individuals had a Mini Mental State Examination score above 26 (Folstein et al. 1975). Hence, with respect to Part II, the final sample for the examination of co-occurring disorders was composed of 138 ASD participants and 170 COM participants.

Based on a tertile split of this ASD group, the participants were assigned to a young (19–38 years), middle-aged (39–54 years), and older (55–79 years) adult group (Table 1). Please note that information about diagnostic status, medical conditions, and medication usage was obtained by means of self-report.

**Table 1:** Descriptives of the ASD and COM group for Part I and II

	ASD				Young versus middle versus older	COM				Young versus middle versus older	ASD versus COM
	All ages	Young	Middle	Older	Fisher’s χ^2^ or *F*	All ages	Young	Middle	Older	Fisher’s χ^2^ or *F*	χ^2^
*Part I*											
N	172	52	72	48		172	60	47	65		
Gender					4.27					1.75	4.45*
Male	116	33	45	38		97	37	23	37		
Female	56	19	27	10		75	23	24	28		
Education^a^					11.98					15.14^+^	9.77^+^
Low	1	0	1	0		0	0	0	0		
Middle	55	18	19	18		37	9	10	18		
High	115	34	51	30		134	51	37	46		
Diagnosis					6.71					–	–
Autistic disorder	26	5	12	9		–	–	–	–		
Asperger	88	27	35	26		–	–	–	–		
PDD-NOS	53	16	24	13		–	–	–	–		
ASD	5	4	1	0		–	–	–	–		
ISCO					19.29**					49.93***	7.70^+^
Class 1–3	62	11	37	14		80	19	36	25		
Class 4–6	21	8	10	3		22	13	5	4		
Class 7–9	11	2	4	5		4	4	0	0		
Unemployed	74	30	20	24		57	23	1	33		
Age (mean)	46.7	29.3	47.9	63.7	525.52***	46.0	26.8	47.0	63.0	711.07***	0.16
AQ (mean)	33.5	32.1	34.4	33.4	1.16	12.4	12.3	11.1	13.0	1.77	831.22***
IQ (mean)	NA	NA	NA	NA		NA	NA	NA	NA		
MMSE (mean)	NA	NA	NA	NA		NA	NA	NA	NA		
ADOS (mean)	NA	NA	NA	NA		–	–	–	–		
Psychotropic medication	87	28	38	21	1.26	6	0	2	4	3.75	96.69***
Antidepressants^b^	52	18	23	11	1.80	4	0	1	3	2.61^+^	49.14***
Anxiolytic/sedative/hypnotics	19	6	8	5	0.10	1	0	0	1	1.61	17.20***
Antipsychotics	24	14	7	3	9.62**	0	0	0	0	–	25.80***
Stimulants	14	4	8	2	1.73	0	0	0	0	–	14.59***
Other psychotropic medication	11	1	8	2	4.22	1	0	1	0	2.25	8.64**
Other non-psychotropic medication	58	9	27	22	10.23**	55	6	15	34	27.04***	0.12
*Part II*											
N	138	46	47	45		170	60	46	64		
Gender					3.83					1.47	5.09*
Male	96	31	29	36		97	37	23	37		
Female	42	15	18	9		73	23	23	27		
Education^a^					10.77					13.49^+^	8.50^+^
Low	1	0	1	0		0	0	0	0		
Middle	43	15	11	17		36	9	9	18		
High	94	31	35	28		134	51	37	46		
Diagnosis					6.82					–	–
Autistic disorder	21	4	9	8		–	–	–	–		
Asperger	69	24	21	24		–	–	–	–		
PDD-NOS	43	14	16	13		–	–	–	–		
ASD	5	4	1	0		–	–	–	–		
ISCO					14.98*					48.84***	7.37^+^
Class 1–3	48	10	25	13		79	19	35	25		
Class 4–6	16	7	6	3		22	13	5	4		
Class 7–9	6	1	1	4		4	4	0	0		
Unemployed	66	27	15	24		57	23	1	33		
Age (mean)	46.5	28.8	47.2	63.9	481.64***	45.9	26.8	47.2	62.9	703.46***	0.11
AQ (mean)	33.5	31.7	35.2	33.4	2.06	12.2	12.3	11.0	13.0	1.83	723.60***
IQ (mean)	113.8	112.1	116.7	112.5	1.10	113.3	111.2	114.1	114.8	0.78	0.06
MMSE (mean)	29.0	28.9	29.1	29.1	0.57	29.1	29.3	29.1	29.0	1.41	0.71
ADOS (mean)	8.6	9.5	8.5	8.0	2.43^+^	–	–	–	–	–	–
Psychotropic medication	67	22	27	18	2.80	6	0	2	4	3.82	85.37***
Antidepressants^b^	38	12	16	10	1.65	4	0	1	3	2.65	41.02***
Anxiolytic/sedative/hypnotics	16	5	6	5	0.17	1	0	0	1	1.62	17.69***
Antipsychotics	18	11	4	3	6.46*	0	0	0	0	–	23.55***
Stimulants	9	4	4	1	2.11	0	0	0	0	–	11.42***
Other psychotropic medication	9	1	7	1	6.73**	1	0	1	0	2.28	8.54**
Other non-psychotropic medication	46	7	20	19	10.73**	53	6	14	33	26.08***	0.16

*ASD* autism spectrum disorder, *COM* comparison group, *PDD-NOS* pervasive developmental disorder not otherwise specified, *ISCO* International Standard Classification of Occupations, *AQ* Autism-Spectrum Quotient, *IQ* estimated intelligence quotient, *MMSE* Mini Mental State Examination, *ADOS* Autism Diagnostic Observation Schedule

^a^One missing in both groups

^b^Antidepressant medication refers to the use of non-selective monoamine reuptake inhibitors, selective serotonin reuptake inhibitors, and other antidepressants

^+^ *p* < .1; * *p* ≤ .05; ** *p* ≤ .01; *** *p* ≤ .001

### Measures

#### Psychiatric Co-occurring Symptoms

##### Symptom Checklist-90 Revised (SCL-90-R)

The SCL-90-R (Arrindell and Ettema 2005; Derogatis 1977) is a widely used multidimensional self-report inventory consisting of 90 items to assess the presence of current psychopathological symptoms and psychological distress. Each item is rated on a five-point Likert scale ranging from 0 “not at all” to 4 “very much” and indicates how much distress was caused during the last week comprising today. The original SCL-90-R includes nine primary symptom dimensions and three global indices that cover clinically relevant psychiatric and psychosomatic symptoms. The Dutch version (Arrindell and Ettema 2005), however, measures eight dimensions: anxiety, agoraphobia, depression, somatization, cognitive-performance deficits, interpersonal sensitivity and mistrust, hostility, and sleep difficulties. The total score, psychoneuroticism, provides a general measure of psychological distress. Higher scores indicate more symptoms and distress. The psychometric properties of the SCL-90-R, including internal consistency, test–retest reliability, and convergent and divergent validity, are good to very good (Arrindell and Ettema 2005).

#### Psychiatric Co-occurring Disorders

##### Mini International Neuropsychiatric Interview Plus (MINI-Plus)

The MINI-Plus (Sheehan et al. 1998; van Vliet et al. 2000) is a structured diagnostic interview that explores several psychiatric disorders according to DSM-IV criteria. First, two to four screenings questions are asked for each disorder. Second, if any of these is answered positively, additional questions further inquire about the presence of a disorder. We inquired about mood, anxiety, substance-related, eating, somatoform, and conduct disorders. The MINI has good inter-rater and test–retest reliability (Lecrubier et al. 1997; Sheehan et al. 1997). For the current study, we adjusted wording of a small number of questions, for example by splitting extended questions into sub questions, to make them more comprehensible to individuals with ASD and to be able of examining lifetime adherence for all disorders. Although we did not change the purport of the items, the validity of the MINI may have been reduced due to these adjustments.

##### ADHD Rating Scale

The ADHD rating scale (Kooij et al. 2005) is a 23-item self-report questionnaire to assess ADHD symptoms based on DSM-IV criteria. Using the adult scale, an individual rates the extent to which each statement illustrates his or her behavior over the past 6 months on a four-point Likert scale, ranging from 0 “rarely or never” to 3 “very often”. Items rated with “often” or “very often” met diagnostic criteria for either inattentive or hyperactive-impulsive subtype symptoms. Following the DSM-IV (American Psychiatric Association 2000), we considered the presence of at least six out of nine symptoms per subtype as indicative of an AD(H)D diagnosis. The validity of the ADHD rating scale is reasonable (Kooij et al. 2008).

#### Risk Factors

ASD severity as measured with the AQ and ADOS, intellectual functioning (IQ) as estimated with a short version of the WAIS-III, general cognitive functioning as measured with the MMSE, and information on education, work situation (coded according to the International Standard Classification of Occupations [ISCO]), living situation, gender, and age as indicated by self-report constituted the factors that were considered as being potentially predictive of psychopathology.

### Procedure

Informed consent was obtained from all individual participants included in the study, after which they filled out the AQ and SCL-90, among other questionnaires (Part I). Participants selected for Part II were tested in two sessions during which (1) the ADOS, shortened WAIS-III, MMSE, and MINI were administered, and (2) neuropsychological and experimental testing took place (these are described elsewhere) (e.g., Lever & Geurts 2015a). Participants who were tested in at least one test session received compensation for their travel expenses; most COM participants also received additional compensation (max €20). The study was approved by the institutional review board of the University of Amsterdam and was in accordance with the 1964 Helsinki declaration and its later amendments or comparable ethical standards.

### Statistical Analyses

#### Psychiatric Co-occurring Symptoms

SCL-90-R variables were highly skewed and neither log, square root, nor inverse transformation lead to normality. However, as MANOVA is thought be robust against this type of violation (Stevens 2012), we ran a MANOVA with Diagnostic group (ASD, COM) and Age (young, middle-aged, older) as between-subject factors and the total score and SCL-90-R subscales as dependent variables. Raw scores were then compared with normative data available for the general population and a policlinic psychiatric patient group (Arrindell and Ettema 2005). Analyses were run with and without outliers (data points more than 3 SD from group mean). When the pattern of results changed by removing outliers, we report both analyses.

#### Psychiatric Co-occurring Disorders

Chi square tests were used to compare frequencies of psychiatric disorders, as measured with the MINI-Plus and ADHD list, between the ASD and COM group. We clustered the inquired disorders into six major disorders: mood, anxiety, substance-related, eating, somatoform, and attentional and behavioral disorders and Bonferroni corrected for multiple comparisons (i.e., significance level was set on 0.05/6 = 0.0083). Thereafter, Chi square tests were ran per non-clustered disorder to compare the ASD and COM group and Fisher’s exact test was used to compare frequencies between young, middle-aged and older adults per diagnostic group. No further correction was applied to these analyses. Results per distinct disorder are presented when group differences were significant after Bonferroni correction. Otherwise, they are presented in the supplementary material (Online Resource 1).

#### Risk Factors

Binomial logistic regressions and linear regressions were run to assess the association between potential risk factors and any mood or anxiety disorder and depression and anxiety symptoms, respectively. Please note that we computed these risk factor analyses on the sample of Part II (due to the inclusion of IQ and ADOS) and that we excluded the COM group from these analyses (as our focus was on risk factors involved in the ASD group). All analyses were conducted in SPSS 22.0 (IBM Corp. 2013).

## Results

### Psychiatric Co-occurring Symptoms

The SCL-90-R scores for Part I are presented in Table 2. The omnibus MANOVA revealed a main effect of diagnostic group, Λ = 0.58, *F*(9, 330) = 26.42, *p* < .001, *η*_p_^2^ = .42, but no main effect of age-group, Λ = 0.96, *F*(18, 660) = 0.82, *p* = .672, *η*_p_^2^ = .02, nor an interaction effect, Λ = 0.94, *F*(18, 660) = 1.15, *p* = .298, *η*_p_^2^ = .03. The ASD group had higher scores on all subscales and the total score. This is in line with the findings when we compare the scores of the ASD sample to the norms of a general population sample as over a quarter of adults with ASD had depression or anxiety scores that were considered very high (≥95th percentile). However, compared to a psychiatric patient group, only a few individuals (<5 %) with ASD had scores above the 95th percentile (Table 3), which suggest that these high scores for individuals with ASD are common in individuals with psychiatric diagnoses.

**Table 2:** SCL-90-R total and subscale scores for the young, middle-aged, and older adults with and without ASD

	ASD				COM				ASD versus COM^a^	
	All ages	Young	Middle	Older	All ages	Young	Middle	Older	*F*	*η*_*p*_^*2*^
Psychoneuroticism	174.9	183.9	173.1	167.6	113.3	111.3	115.9	113.2	192.96***	.36
Agoraphobia	11.4	12.2	11.3	10.6	7.4	7.3	7.2	7.5	116.89***	.26
Anxiety	18.3	19.8	18.1	17.0	11.8	11.4	12.1	11.8	111.28***	.25
Depression	33.6	34.8	33.9	31.8	20.6	19.8	21.1	21.0	151.71***	.31
Somatization	20.5	21.6	20.8	18.9	15.3	15.4	15.5	15.1	62.81***	.16
Cognitive-performance deficits	21.1	21.7	21.1	20.5	12.5	12.7	12.7	12.2	208.91***	.38
Interpersonal sensitivity and mistrust	37.2	38.8	36.1	37.2	23.4	22.7	24.3	23.3	152.44***	.31
Hostility	9.9	10.8	9.5	9.3	7.0	7.1	7.2	6.8	83.57***	.20
Sleep difficulties	6.6	6.9	6.7	6.2	4.7	4.6	4.7	4.8	43.21***	.11

*ASD* autism spectrum disorder, COM comparison group

^a^We do not report the effects of age-group as the overall MANOVA revealed a nonsignificant effect, as denoted in the main text

**Table 3:** Number (%) of adults with and without ASD scoring above the 95th percentile compared to a normative general population and a psychiatric patient sample

	ASD		COM	
	NOR	PSY	NOR	PSY
Psychoneuroticism	69 (40.1 %)	3 (1.7 %)	5 (2.9 %)	0 (–)
Agoraphobia	79 (45.9 %)	1 (0.8 %)	2 (1.2 %)	0 (–)
Anxiety	44 (25.6 %)	1 (0.8 %)	2 (1.2 %)	0 (–)
Depression	70 (40.7 %)	1 (0.8 %)	5 (2.9 %)	0 (–)
Somatization	29 (16.9 %)	3 (1.7 %)	3 (1.7 %)	0 (–)
Cognitive-performance deficits	87 (50.6 %)	5 (2.9 %)	6 (3.5 %)	0 (–)
Interpersonal sensitivity and mistrust	67 (39.0 %)	8 (4.7 %)	9 (5.2 %)	0 (–)
Hostility	51 (29.7 %)	3 (1.7 %)	6 (3.5 %)	0 (–)
Sleep difficulties	42 (24.4 %)	4 (2.3 %)	10 (5.8 %)	0 (–)

*ASD* autism spectrum disorder, *COM* comparison group, *NOR* general population; *PSY* psychiatric patient sample

When running the MANOVA on the subgroup sample of Part II, there was still a main effect of diagnostic group and no main effect of age-group, but now the diagnostic group by age-group interaction was significant, Λ = 0.88, *F*(18, 588) = 2.16, *p* = .004, *η*_p_^2^ = .06, with generally a decrease of reported symptoms with age in the ASD group and no such a decrease in the COM group. After removing the outliers, in addition to the already present effects, there was also a main effect of age-group, Λ = 0.88, *F*(18, 542) = 1.95, *p* = .011, *η*_p_^2^ = .06. The older age group generally had lower scores than the younger groups, even though this difference seemed more pronounced in the ASD group.

### Psychiatric Co-occurring Disorders

The frequencies of the investigated lifetime DSM-IV diagnoses are presented in Table 4. In the ASD group, 79.0 % met one or more lifetime diagnosis for a psychiatric disorder against 48.8 % of the COM group. Overall, older adults with ASD less often met diagnostic criteria compared to the younger age groups, whereas there were no differences between age groups among adults without ASD. In the ASD group, while 21 % did not meet criteria for any psychiatric diagnoses and 20.3 % met criteria for one psychopathology, over 57 % had more than one co-occurring lifetime disorder. In the COM group, the large majority did meet criteria for one or none lifetime DSM-IV diagnosis. Nevertheless, a small percentage (15.9 %) of the individuals without ASD had more than one co-occurring psychopathology (Table 5).

**Table 4:** Lifetime rates of DSM-IV disorders in young, middle-aged, and older adults with and without ASD

	ASD									COM									
	All ages		Young		Middle		Older		Young versus middle versus older	All ages		Young		Middle		Older		Young versus middle versus older	ASD versus COM
	N	%	N	%	N	%	N	%	Fisher’s χ^2^	N	%	N	%	N	%	N	%	Fisher’s χ^2^	χ^2^
Any psychiatric disorder	109	79.0	38	82.6	41	87.2	30	66.7	6.02*	83	48.8	30	50.0	19	41.3	34	53.1	1.55	29.52***
Mood disorders	79	57.2	24	52.2	35	74.5	20	44.4	9.30**	31	18.2	9	15.0	9	19.6	13	20.3	0.70	50.49***
Depression	74	53.6	19	52.2	31	66.0	19	42.2	5.24^+^	28	16.5	9	15.0	8	17.4	11	17.2	0.20	47.47***
Dysthymia	25	18.1	6	13.0	13	27.7	6	13.3	4.04	5	2.9	1	1.7	2	4.3	2	3.1	0.86	19.95***
PDysD (only females)	9	20.9	3	18.8	3	16.7	3	33.3	5.16	2	2.7	1	4.3	1	4.3	0	–	3.00	14.34***
Anxiety disorders	74	53.6	30	65.2	25	53.2	19	42.2	4.81^+^	25	14.7	8	13.3	8	17.4	9	14.1	0.44	52.89***
Panic disorder	21	15.2	11	23.9	6	12.8	4	8.9	4.01	6	3.5	3	5.0	1	2.2	2	3.1	0.68	13.01***
Agoraphobia	29	21.0	10	21.7	9	19.1	10	22.2	0.20	6	3.5	1	1.7	3	6.5	2	3.1	1.77	23.12***
Social phobia	21	15.2	10	21.7	10	21.3	1	2.2	10.23**	8	4.7	3	5.0	3	6.5	2	3.2	0.86	9.87**
Specific phobia	16	11.6	5	10.9	7	14.9	4	8.9	0.84	8	4.7	1	1.7	1	2.2	6	9.4	4.11	5.03*
PTSS	4	2.9	1	2.2	3	6.4	0	–	4.66	1	0.6	0	–	1	2.2	0	–	2.28	6.36*
OCD	30	21.7	13	28.3	10	21.3	7	15.6	5.85	1	0.6	0	–	1	2.2	0	–	2.28	40.68***
GAD	22	15.9	8	17.4	9	19.1	5	11.1	3.12	5	2.9	2	3.3	2	4.3	1	1.6	1.00	17.50***
Substance-related disorders	22	15.9	9	19.6	5	10.6	8	17.8	1.60	43	25.3	20	33.3	9	19.6	14	21.9	3.10	4.00*
Eating disorders	8	5.8	4	8.7	3	6.4	1	2.2	1.79	1	0.6	1	1.7	0	–	0	–	1.74	7.29*
Somatoform disorders	8	5.8	6	13.0	2	4.3	0	–	6.72*	3	1.8	0	–	1	2.2	2	3.1	1.81	3.60^+^
Attentional and behavioral disorders	43	31.2	14	30.4	16	34.0	13	28.9	0.33	9	5.3	5	8.3	2	4.3	2	3.1	1.65	36.31***
ADHD^a^	42	30.4	14	30.4	15	31.9	13	28.9	0.13	9	5.3	5	8.3	2	4.3	2	3.1	1.65	34.84***
Inattentive	14	10.1	5	10.9	3	6.4	6	13.3	1.30	4	2.4	3	5.0	1	2.2	0	–	3.05	8.40**
Hyperactivity/impulsivity	18	13.0	6	13.0	8	17.0	4	8.9	1.33	5	2.9	2	3.3	1	2.2	2	3.1	0.32	11.25***
Combined	10	7.2	3	6.5	4	8.5	3	6.7	0.28	0	–	0	–	0	–	0	–	–	12.73***
Conduct disorder	3	2.2	1	2.2	2	4.3	0	–	4.46	1	0.6	0	–	1	2.2	0	–	2.28	4.35

*ASD* autism spectrum disorder, *COM* comparison group, *PDysD* premenstrual dysphoric disorder, *PTSS* post-traumatic stress disorder, *OCD* obsessive compulsive disorder, *GAD* generalized anxiety disorder, *ADHD* attention deficit hyperactivity disorder

^+^ *p* < .1; * *p* ≤ .05; ** *p* ≤ .01; *** *p* ≤ .001

^a^Measured with the ADHD list instead of the Mini International Neuropsychiatric Interview Plus. Please note that we used the presence of an AD(H)D diagnosis as an exclusion criterion in the COM group, based on which three individuals were excluded. Hence, this prevalence rate is likely an underestimation

**Table 5:** Frequencies and percentages of the number of lifetime diagnoses in young, middle-aged, and older adults with and without ASD

	ASD								COM							
	All ages		Young		Middle		Older		All ages		Young		Middle		Older	
	N	%	N	%	N	%	N	%	N	%	N	%	N	%	N	%
No DSM-IV diagnoses	29	21.0	8	17.4	6	12.8	15	33.3	87	51.2	30	50.0	27	58.7	30	46.9
1 DSM-IV diagnosis	28	20.3	6	13.0	13	27.7	9	20.0	56	32.9	16	26.7	13	28.3	27	42.2
2 DSM-IV diagnoses	19	13.8	8	17.4	7	14.9	4	8.9	16	9.4	8	13.3	3	6.5	5	7.8
3 DSM-IV diagnoses	24	17.4	8	17.4	6	12.8	10	22.2	4	2.4	2	3.3	1	2.2	1	1.6
4 DSM-IV diagnoses	17	12.3	9	19.6	3	6.4	5	11.1	2	1.2	2	3.3	0	–	0	–
>4 DSM-IV diagnoses	21	15.2	7	15.2	12	25.5	2	4.4	5	2.9	2	3.3	2	4.3	1	1.6

*ASD* autism spectrum disorder, *COM* comparison group

As expected, in adults with ASD, mood disorders were the most common group of psychiatric disorders (57.2 %) and included major depression (53.6 %) and dysthymia (18.1 %). Mood disorders were most prevalent in middle-aged adults and least prevalent in the oldest age-group with ASD. All mood disorders were more frequent in adults with ASD than in adults without ASD. There were no differences between age-groups in the COM group.

The second most common group of disorders in the ASD group were the anxiety disorders (53.6 %) of which obsessive–compulsive disorder (OCD; 21.7 %) and agoraphobia (21.0 %) most often occurred. The prevalence of any anxiety disorder appeared slightly lower in older adults, but it was not statistically significant. Whereas social phobia was common in young and middle-aged adults, it was not in older adults with ASD. All anxiety disorders were more frequent in adults with ASD than in adults without ASD. In the COM group, there were no differences between age-groups.

**Fig. 1:**
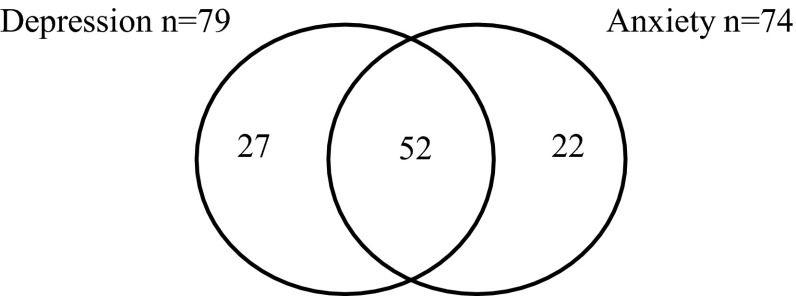
Number of ASD participants showing overlap between mood and anxiety disorders

As mood and anxiety disorders often co-occur (Beekman et al. 2000; Sartorius et al. 1996), we explored the overlap between these two lifetime diagnoses (Fig. 1). Over 65 % of the adults with ASD meeting criteria for any lifetime mood or anxiety disorder, also met criteria for the other co-occurring disorder.

### Associations Between Potential Risk Factors and Mood and Anxiety Symptoms

ASD severity by both self-report and ADOS was predictive of the amount of depression and anxiety symptoms as measured with the SCL-90-R. None of the other factors was significantly associated with these symptoms (Table 6).

**Table 6:** Risk factors associated with depression and anxiety symptoms (SCL-90-R) in adults with ASD

	Depression				Anxiety			
	B	SE	t	95 % CI	B	SE	t	95 % CI
Age	−0.05	0.07	−0.75	−0.18 to 0.08	−0.10	0.06	−1.62	−0.23 to 0.02
Gender	4.24	2.33	1.82	−0.37 to 8.84	3.85	2.25	1.71	−0.61 to 8.31
Education	−1.98	2.23	−0.89	−6.40 to 2.44	−3.04	2.16	−1.41	−7.31 to 1.24
Living situation	−0.53	1.06	−0.50	−2.63 to 1.58	−0.93	1.03	−0.91	−2.97 to 1.10
ISCO	0.05	0.73	0.07	−1.38 to 1.49	0.43	0.70	0.62	−0.96 to 1.83
IQ	−0.06	0.07	−0.93	−0.19 to 0.07	0.01	0.06	0.18	−0.11 to 0.14
MMSE	−0.57	1.04	−0.55	−2.63 to 1.49	−1.39	1.01	−1.38	−3.39 to 0.60
AQ	0.45	0.12	3.62***	0.20 to 0.69	0.40	0.12	3.33***	0.16 to 0.63
ADOS	0.89	0.34	2.66**	0.23 to 1.56	0.77	0.33	2.38*	0.13 to 1.42
Constant	36.94	28.45	1.30	−19.37 to 93.26	57.94	27.54	2.10*	3.44 to 112.45
R^2^	20.5 %				20.9 %			
N	134				134			

*SCL-90-R* Symptom Checklist 90 revised, *ISCO* International Standard Classification of Occupations, *IQ* estimated intelligence quotient, *MMSE* Mini Mental State Examination, *AQ* Autism-spectrum Quotient, *ADOS* Autism Diagnostic Observation Schedule

* *p* ≤ .05; ** *p* ≤ .01; *** *p* ≤ .001

### Associations Between Potential Risk Factors and any Mood and Anxiety Disorder

Female gender was a significant predictor of any mood disorder. Lower age and more severe ASD as indicated by self-report were associated with the presence of any anxiety disorder. None of the other factors was associated with any mood or anxiety disorder (Table 7).

**Table 7:** Risk factors associated with any mood and anxiety disorder (MINI-Plus) in adults with ASD

	Any mood disorder				Any anxiety disorder			
	B	SE	Odds Ratio	95 % CI	B	SE	Odds Ratio	95 % CI
Age	−0.00	0.01	1.00	0.97 to 1.02	−0.04	0.02	0.97*	0.94 to 1.00
Gender	1.46	0.49	4.29**	1.65 to 11.18	0.85	0.52	2.33	0.85 to 6.42
Education	−0.14	0.44	0.87	0.37 to 2.04	−0.67	0.50	0.51	0.19 to 1.37
Living situation	0.18	0.21	1.20	0.80 to 1.80	0.04	0.23	1.05	0.67 to 1.64
ISCO	−0.14	0.14	0.87	0.66 to 1.15	0.09	0.16	1.09	0.80 to 1.48
IQ	−0.00	0.01	1.00	0.97 to 1.02	−0.01	0.01	0.99	0.96 to 1.02
MMSE	−0.03	0.20	0.97	0.66 to 1.43	0.00	0.23	1.00	0.65 to 1.56
AQ	0.01	0.02	1.01	0.97 to 1.06	0.15	0.03	1.16***	1.09 to 1.24
ADOS	0.02	0.07	1.02	0.90 to 1.16	0.04	0.07	1.04	0.90 to 1.20
Constant	−0.56	5.42	0.57		−2.18	6.07	0.11	
N	134				134			

*MINI-Plus* Mini International Neuropsychiatric Interview, *ISCO* International Standard Classification of Occupations, *IQ* estimated intelligence quotient, *MMSE* Mini Mental State Examination, *AQ* Autism-spectrum Quotient, *ADOS* Autism Diagnostic Observation Schedule

* *p* ≤ .05; ** *p* ≤ .01; *** *p* ≤ .001

## Discussion

In the current study, we examined psychiatric symptoms and disorders in young, middle-aged, and older adults with ASD and focused on the two most frequently occurring diagnoses (i.e., mood and anxiety) by testing several potential risk factors covering different domains. As expected, adults with ASD experienced more psychological symptoms and distress compared to a typically developing comparison group. These elevated levels were not only reported by older adults (for similar findings see van Heijst and Geurts 2014), but were consistently high also in young and middle-aged adults and, thus, across the adult lifespan. Whereas at least a quarter of the adults with ASD reported symptoms within the clinical range compared to a population-based sample, only a few participants scored within the clinical range when compared to a psychiatric patient group (see also Joshi et al. 2013). These findings indicate that, as expected, adults with ASD experience many feelings of depression, anxiety, and psychological distress, but this was not atypical when compared to other psychiatric patients.

Consistent with the experience of many psychological symptoms, is the high proportion of individuals meeting criteria for a psychiatric diagnosis. Seventy-nine percent of the adults with ASD have experienced any psychiatric disorder once in their lives. As predicted, most common disorders were mood (57 %) and anxiety disorders (54 %), which often co-occur. ADHD frequently occurred as well (30 %) and notable is the high percentage of females meeting criteria for a premenstrual dysphoric disorder (21 %). The estimated occurrences of psychiatric disorders in a large group of adults with ASD is comparable to those previously reported in other studies of adults without IDs using the Structured Clinical Interview for DSM-IV axis I Disorders (SCID-I) or a structured DSM-IV based clinical interview (Hofvander et al. 2009; Lugnegård et al. 2011; Roy et al. 2015). The MINI is based on both the DSM-IV and ICD-10 (Lecrubier et al. 1997) and the MINI and SCID-I are well concordant with each other (Sheehan et al. 1997). Given the consistency with previous studies involving a similar population, the current findings seem to reflect the true lifetime psychiatric problems of adults with ASD.

However, while others focused on young and middle-aged adults, we also examined older adults and we found that, also in late adulthood, psychiatric disorders were still common. Nevertheless, lifetime diagnoses for any psychiatric disorder were less often present in older than in younger adults with ASD, suggesting reduced psychopathology in late adulthood, a pattern that has been commonly observed in large typical aging studies (Bijl et al. 1998; Kessler et al. 2005). Although a recent study found the opposite (i.e., psychopathology was more common in older than in younger adults) in older adults with ASD and without ID (Roy et al. 2015), this seems mainly due to the inclusion of middle-aged adults in the “older” adult group (age range 40–62 years) in the study of Roy and colleagues. Especially in mid adulthood, psychiatric disorders such as depression seem more common than in older or younger individuals (Bijl et al. 1998; Kessler et al. 2005). While our older adult group consisted of participants until 79 years of age, participants in the Roy study were rather middle-aged, which would explain why high rates were found and why our findings were apparently discordant. We also observed that only one (2 %) older adult met criteria for social phobia (i.e., social anxiety disorder) against 21 % of young and middle-aged adults. Of course, this could be due to a cohort effect. However, there are several potential alternative explanations for this latter finding. First, social phobia and social skills may reciprocally influence each other: individuals with poor social skills may be more likely to experience anxiety related to social interactions, but, inversely, individuals with social anxiety may less likely develop and practice their social skills (Bellini 2004). In fact, adults with social anxiety disorder report difficulties in social skills, similarly to ASD individuals (Cath et al. 2008). Although social symptoms tend to remain stable over time in ASD (Magiati et al. 2014), social functioning seems to improve (Bastiaansen et al. 2011b). Older adults would be more able to adjust their behavior to social situations and cope with their social difficulties, which could have a positive effect on feeling more comfortable in social situations and a negative effect on feelings of anxiety. Second, reduced social anxiety can be associated with a decrement in awareness or concern about social situations, for example due to lower empathic skills (Bellini 2004). However, neither empathic concern (Lever and Geurts 2015b) nor theory of mind (Lever and Geurts 2015a) declined in older adults with ASD, suggesting that this explanation does not hold. Third, older adults may have accepted their difficulties in social situations and, therefore, show less preoccupation and anxiety. Finally, it could be that older adults experience feelings of anxiety in social situations that are qualitatively different than aspects captured by this type of assessment, for example due to differential social settings and type of interactions (Ciliberti et al. 2011). Future research is needed to test which of these potential explanations will hold.

In line with previous studies in adults with ASD (García-Villamisar and Rojahn 2015; Sterling et al. 2008), individuals with more depression and anxiety symptoms also demonstrated more severe self-reported and observed ASD symptoms. When focusing on psychiatric disorders rather than symptoms, higher self-reported ASD symptomatology and lower age were associated with the presence of any lifetime anxiety disorder. This latest result confirmed the already observed trend in the age-group comparisons. Furthermore, female gender was associated with any lifetime mood disorder, indicating that females are more likely to receive a diagnosis of depression or dysthymia than males. Although in line with observations in the general population (Kessler et al. 2005), no such gender differences have been detected in previous adult ASD studies (Lai et al. 2011; Lugnegård et al. 2011). The use of self-report information (Lai et al. 2011) or the inclusion of young adults (Lugnegård et al. 2011) may account for this discrepancy. As aforementioned we did not find a relation between depressive symptoms and gender by means of self-report either and when we (post hoc) selected only young adults within our sample we also did not observe a gender difference on mood disorder. Hence, our findings do suggest that after young adulthood females with ASD are more vulnerable for mood disorder than males with ASD, just as reported in the general population. The other variables (i.e., intellectual and general cognitive functioning, social economic status [education and work], and living situation), selected for their consistent relationship with psychopathology in the general population, were notably not associated with depression and anxiety symptoms and disorders in the ASD group.

Our study suffers from a few limitations that are of importance to keep in mind when interpreting the findings. First, we did neither include an epidemiological sample nor did we adopt a longitudinal design. Therefore, our results can be an overestimation of prevalence rates (Howlin and Moss 2012) and cohort effects can bias our results. Within the current design the directionality of effect cannot be determined: For example, does more severe ASD symptoms cause more psychiatric problems, or is more severe ASD inherently related to psychopathology? Longitudinal research may shed light on this issue. Second, the structured nature of the MINI interview did not allow disentangling whether specific symptoms were characteristic of the investigated disorder or part of the ASD phenotype (e.g., OCD or social anxiety) (see Kerns and Kendall 2012; Wood and Gadow 2010). A promising approach that might shed light on the problem of defining diagnostic boundaries is presented by the network perspective in which mental disorders are conceptualized as networks of interacting symptoms rather than a latent construct (e.g., see Borsboom et al. 2011; Bringmann et al. 2013; Cramer et al. 2010). Third, although prevalence rates of psychiatric disorders in adults without ASD are largely comparable to those obtained in epidemiological studies including a general population sample (Bijl et al. 1998; Kessler et al. 2005), the frequency of substance-related disorders was high. This is mainly due to the prevalence of alcohol abuse (see Online Resource 1). Fourth, diagnostic information was obtained by means of self-report, even though we administered the ADOS to part of the ASD sample to substantiate ASD diagnoses. Yet, given the finding that group differences on a continuous measure of psychopathology were also present in this subsample, it is unlikely that our sampling method affected the main conclusions. Fifth, our findings apply to adults with ASD without ID and cannot be generalized to the entire ASD population. Finally, although we investigated many psychiatric disorders, we did not examine all. For example, schizophrenia, bipolar disorder, and personality disorders were not taken into consideration. Furthermore, we did not focus on medical comorbidities, even though we did collect information regarding the use of non-psychotropic medication. While the percentages of prescribed psychotropic drugs are in line with the high number of observed psychiatric diagnoses, the percentages of non-psychotropic medication use in the ASD and COM group were similar. This might suggest that there are no differences between groups with regard to medical conditions, but this would be a premature conclusion. Those with ASD might report less somatic complaints to their general practitioner due to reduced sensitivity to bodily signals or they might be more reluctant to access the healthcare system due to, for example, communication and social difficulties or anxiety for medical examination as a result of sensory sensitivities. Earlier studies focusing on medical conditions in ASD reported elevated rates compared to controls on many disorders, including epilepsy, gastrointestinal and sleep disorders, diabetes, and dyslipidemia (Croen et al. 2015; Kohane et al. 2012; Tyler et al. 2011). Hence, in future research it would be worthwhile not to merely focus on psychiatric comorbidities but also on somatic comorbidities.

To conclude, in this large ASD adult cohort study including older adults, we showed that psychopathology, and specifically social phobia, less frequently occurred in late adulthood. As these findings represent just an initial step into the understanding of psychopathology across the entire adult lifespan, further research into the nature of psychiatric co-occurring symptoms and disorders and intricate risk factors in old age is needed. Given that psychiatric problems are, however, still common and psychological distress is substantial, we need adequate interventions and support to reduce the personal burden of adults with ASD.

## Electronic supplementary material

Below is the link to the electronic supplementary material.

Supplementary material 1 (DOCX 20 kb)

## Footnotes

1 Please note that the Dutch multidisciplinary guidelines (Trimbos 2013), based on the UK guidelines (National Institute for Health and Clinical Excellence 2012), for diagnosis and treatment of ASD in adults recommends that ASD is diagnosed by a psychiatrist or psychologist experienced in diagnosing ASD who is embedded within a multidisciplinary team, that review of developmental history is necessary (but can be left out when unreliable, despite several attempts to obtain it and when there are well-founded other clinical indications of ASD being concerned), that multiple informants should be asked about current functioning, and that assessment of co-existing conditions should be included.
