# Supplementary material for: Psychiatric Co-occurring Symptoms and Disorders in Young, Middle-Aged, and Older Adults with Autism Spectrum Disorder
Source: J Autism Dev Disord. 2016 Feb 9;46:1916–30. doi: 10.1007/s10803-016-2722-8 (PMC4860203; doi:10.1007/s10803-016-2722-8)
Supplement: Supplementary file 1 — Supplementary material 1 (DOCX 20 kb) [file 10803_2016_2722_MOESM1_ESM.docx]

**Supplementary material**

Lever, A. G., & Geurts, H. M. (2016). Psychiatric co-occurring symptoms and disorders in young, middle-aged, and older adults with autism spectrum disorder. *Journal of Autism and Developmental Disorders.*

Corresponding author:

Anne G. Lever, Msc, Dutch Autism & ADHD Research Center, Department of Psychology, University of Amsterdam, Nieuwe Achtergracht 129B, 1018 WT Amsterdam, The Netherlands, e: A.G.Lever@uva.nl, p: +31 20 525 6887, f: +31 20 639 1656.

**Table 1.** Lifetime rates of substance-related, eating, and somatoform DSM-IV disorders in young, middle-aged, and older adults with and without ASD.

|  | ASD | |  | |  | |  | |  | COM | |  | |  | |  | |  |  |
| --- | --- | --- | --- | --- | --- | --- | --- | --- | --- | --- | --- | --- | --- | --- | --- | --- | --- | --- | --- |
|  | All ages | | Young | | Middle | | Older | |  | All ages | | Young | | Middle | | Older | |  | ASD vs COM |
|  | N | % | N | % | N | % | N | % | Fisher’s χ^2^ | N | % | N | % | N | % | N | % | Fisher’s χ^2^ | χ^2^ |
| Substance-related disorders | 22 | 15.9 | 9 | 19.6 | 5 | 10.6 | 8 | 17.8 | 1.60 | 43 | 25.3 | 20 | 33.3 | 9 | 19.6 | 14 | 21.9 | 3.10 | 4.00* |
| Alcohol abuse | 19 | 13.8 | 7 | 15.2 | 5 | 10.6 | 7 | 15.6 | 4.66 | 41 | 24.1 | 19 | 31.7 | 9 | 19.6 | 13 | 20.3 | 4.28 | 8.31* |
| Alcohol dependence | 7 | 5.1 | 1 | 2.2 | 4 | 8.5 | 2 | 4.4 | 1.82 | 7 | 4.1 | 4 | 6.7 | 0 | - | 3 | 4.7 | 2.97 | 0.16 |
| Drugs abuse | 6 | 4.3 | 4 | 8.7 | 1 | 2.1 | 1 | 2.2 | 5.07 | 11 | 6.5 | 10 | 16.7 | 0 | - | 1 | 1.6 | 18.13*** | 4.07 |
| Drugs dependence | 4 | 2.9 | 3 | 6.5 | 1 | 2.1 | 0 | - | 2.93 | 7 | 4.1 | 7 | 11.7 | 0 | - | 0 | - | 12.81*** | 1.16 |
| Eating disorders | 8 | 5.8 | 4 | 8.7 | 3 | 6.4 | 1 | 2.2 | 1.79 | 1 | 0.6 | 1 | 1.7 | 0 | - | 0 | - | 1.74 | 7.29* |
| Anorexia nervosa | 5 | 3.6 | 3 | 6.5 | 2 | 4.3 | 0 | - | 4.69 | 0 | - | 0 | - | 0 | - | 0 | - | - | 6.29* |
| Bulimia nervosa | 3 | 2.2 | 1 | 2.2 | 1 | 2.1 | 1 | 2.2 | 2.27 | 1 | 0.6 | 1 | 1.7 | 0 | - | 0 | - | 1.74 | 2.75 |
| Somatoform disorders | 8 | 5.8 | 6 | 13.0 | 2 | 4.3 | 0 | - | 6.72* | 3 | 1.8 | 0 | - | 1 | 2.2 | 2 | 3.1 | 1.81 | 3.60+ |
| Somatization | 4 | 2.9 | 4 | 8.7 | 0 | - | 0 | - | 7.83** | 0 | - | 0 | - | 0 | - | 0 | - | - | 6.26* |
| Pain disorder | 3 | 2.2 | 1 | 2.2 | 2 | 4.3 | 0 | - | 3.61 | 3 | 1.8 | 0 | - | 1 | 2.2 | 2 | 3.1 | 1.81 | 1.31 |
| Hypochondriasis | 1 | 0.7 | 1 | 2.2 | 0 | - | 0 | - | 3.55 | 0 | - | 0 | - | 0 | - | 0 | - | - | 2.48 |
| BDD | 1 | 0.7 | 1 | 0.9 | 0 | - | 0 | - | 3.69 | 0 | - | 0 | - | 0 | - | 0 | - | - | 2.48 |

*Note*. ASD = autism spectrum disorder; COM = comparison group; BDD = body dysmorphic disorder.

^+^*p*<.1, **p*≤.05, ***p*≤.01, ****p*≤.001
